# Microenvironment-Driven Dynamic Heterogeneity and Phenotypic Plasticity as a Mechanism of Melanoma Therapy Resistance

**DOI:** 10.3389/fonc.2018.00173

**Published:** 2018-05-24

**Authors:** Farzana Ahmed, Nikolas K. Haass

**Affiliations:** ^1^The University of Queensland Diamantina Institute, Translational Research Institute, The University of Queensland, Brisbane, QLD, Australia; ^2^Discipline of Dermatology, University of Sydney, Sydney, NSW, Australia

**Keywords:** melanoma, tumor heterogeneity, clonality, cancer drug resistance, tumor microenvironment, microphthalmia-associated transcription factor, tumor plasticity, slow-cycling tumor cells

## Abstract

Drug resistance constitutes a major challenge in designing melanoma therapies. Microenvironment-driven tumor heterogeneity and plasticity play a key role in this phenomenon. Melanoma is highly heterogeneous with diverse genomic alterations and expression of different biological markers. In addition, melanoma cells are highly plastic and capable of adapting quickly to changing microenvironmental conditions. These contribute to variations in therapy response and durability between individual melanoma patients. In response to changing microenvironmental conditions, like hypoxia and nutrient starvation, proliferative melanoma cells can switch to an invasive slow-cycling state. Cells in this state are more aggressive and metastatic, and show increased intrinsic drug resistance. During continuous treatment, slow-cycling cells are enriched within the tumor and give rise to a new proliferative subpopulation with increased drug resistance, by exerting their stem cell-like behavior and phenotypic plasticity. In melanoma, the proliferative and invasive states are defined by high and low microphthalmia-associated transcription factor (MITF) expression, respectively. It has been observed that in MITF^high^ melanomas, inhibition of MITF increases the efficacy of targeted therapies and delays the acquisition of drug resistance. Contrarily, MITF is downregulated in melanomas with acquired drug resistance. According to the phenotype switching theory, the gene expression profile of the MITF^low^ state is predominantly regulated by WNT5A, AXL, and NF-κB signaling. Thus, different combinations of therapies should be effective in treating different phases of melanoma, such as the combination of targeted therapies with inhibitors of MITF expression during the initial treatment phase, but with inhibitors of WNT5A/AXL/NF-κB signaling during relapse.

## Introduction

The development of targeted therapies for metastatic melanoma using small molecule MAPK pathway inhibitors (MAPKi) or immune checkpoint antagonists (ICi) has revolutionized dermatological oncology. However, first-generation MAPKi only works in approximately 35–50% of cases as a BRAF^V600^ mutation must be present (1, 2). ICi show response rates of up to 60%, depending on drug or combination, and many of these are durable effects (3). Yet, drug resistance constitutes a major challenge for effective cancer treatment with melanoma being no exception. Rapid resistance to MAPKi is common and has also been reported for ICi (4–9). Although the molecular mechanisms leading to inherent and acquired drug resistance have been discussed extensively in the literature, the dynamics leading to resistance are poorly understood but yet critical to designing better treatments. Besides genetic and epigenetic factors, other contributors to drug resistance are microenvironment-driven tumor heterogeneity and plasticity (10–16).

## Mechanisms of Intrinsic and Acquired Drug Resistance in Melanoma

Intrinsic refers to a pre-existent drug resistance of the entire population or a subpopulation of cancer cells before exposure to the drug. For example, intrinsically resistant cancer cells do not harbor the targeted mutation or are not dependent on the pathway inhibited by the drug. In the case of acquired drug resistance, the tumor responds initially to the treatment but relapses and progresses later. However, it is difficult to distinguish between intrinsic and acquired resistance as a small subpopulation of intrinsically resistant cancer cells subsequently enriched, may also explain initial response and later relapse (17–20). Causative factors that contribute to MAPKi resistance can be broadly classified into three categories: mutational events, non-mutational events, and changes in the surrounding microenvironment (21). Mutational and non-mutational events that contribute to the development of drug resistance have been discussed previously (21, 22) and are not the focus of this review. In brief, the mechanisms linked with these events predominantly lead to MAPK pathway reactivation and/or activation of parallel signaling pathways (e.g., PI3K/AKT/mTOR) (21, 23). Besides mutational and non-mutational events which are intrinsic to tumor cells, the tumor microenvironment contributes to the development of drug resistance by influencing the crosstalk between distinct cellular compartments. Solid tumors are comprised of tumor cells and stromal cells (e.g., fibroblasts, endothelial cells, and lymphocytes) that form an organ-like structure which is embedded within the extracellular matrix (ECM) and nourished by a vascular network. Each of these components show varying distribution within the tumor resulting in a highly complex and heterogeneous tumor microenvironment (24). In melanoma, secretion of tumor necrosis factor-α (25, 26), hepatocyte growth factor (HGF) (27), Wnt antagonist, sFRP2 (28), and increased production of ECM (29) by stromal cells in the tumor can cause resistance to MAPKi. Thus, the density of stromal cells in different parts of the tumor plays a key role in determining response and resistance to MAPKi. In addition, the distribution of the vasculature plays a crucial role in the acquisition of varying drug resistance mechanisms in different parts of the tumor, due to differences in the levels of nutrients and oxygen. Hypoxia can induce resistance to MAPKi by mediating upregulation of HGF/MET signaling (30), increasing SNAIL, and decreasing E-cadherin expression (31).

## Tumor Heterogeneity and Plasticity

Tumor heterogeneity refers to the presence of subpopulations of cells that differ phenotypically and/or by biological behavior, either within a tumor (intra-tumoral) or between tumors of the same histopathological subtype within a patient (inter-tumoral) or between patients (inter-patient) (32). Melanoma heterogeneity plays a key role in the response to MAPKi (5, 20). At the molecular level, the features of different subpopulations are conferred by alterations of the genome, transcriptome, epigenome, and proteome (33, 34). Melanoma is one of the most heterogeneous cancers (35), harboring diverse genomic alterations, including gain of function mutations (e.g., *NRAS, BRAF, KIT, CDK4*, and *MITF*), loss of function mutations (e.g., *CDKN2A, PTEN, ARID2*, and *NF*), and epigenetic changes (e.g., *PTEN, CDKN2A, RAC1*, and *P53*) (36). In addition, various biological markers of melanoma (e.g., CD20, CD133, ABCB5, CD271, JARID1B, and ALDH1) show differential expression patterns in different regions within a tumor (36).

There are three tumor heterogeneity models (37). The well-accepted clonal evolution model (38) refers to acquired additional genetic mutations in cancer cells that contribute to their altered phenotype and malignant potential. This results in a Darwinian-style selection of clones during disease progression (38). The stem cell model suggests that only a small fraction of tumor cells have the potential for maintaining the tumor and drive progression (39). These cancer stem cells have self-renewal capability and can be differentiated into “non-stem cancer cells” that lose their tumorigenic potential by acquiring stable epigenetic changes and occupy the largest fraction of the tumor (37, 39, 40). These two models are complementary to each other, rather than mutually exclusive (41). Their common feature is the unidirectional, irreversible nature of the molecular changes that lead to tumor heterogeneity (37). An alternative model is “phenotypic plasticity” or “phenotype switching.” This model suggests that tumor cells with different phenotypic and functional behavior can dynamically shift between different transcriptional programs (42–44). The different phenotypic states, described in terms of differential gene expression patterns, have been termed “proliferative” and “invasive” signatures (45). In this model, molecular changes resulting in tumor heterogeneity are reversible, unlike the clonal evolution and stem cell models. These changes are predominantly regulated by cues from the surrounding microenvironment, e.g., hypoxia, stroma-derived factors like HGF, TGF-β. For example, in response to hypoxia, proliferative melanoma cells can switch to the invasive phenotype by altering their gene expression profile (10, 46).

## Microenvironment-Driven Dynamic Heterogeneity in Melanoma

“Tumor microenvironment” is a broad term, which includes (1) the tumor stroma composed of fibroblasts, endothelial cells, immune cells, soluble molecules, and the ECM, (2) the epidermal microenvironment where the tumor had originated from, and (3) different subcompartments within the tumor itself (47). Interactions between tumor cells and the microenvironment contribute to the malignant behavior of tumor cells, e.g., progression, metastasis, angiogenesis, migration, and invasion (48, 49). In addition, microenvironmental stress signals in response to nutrient starvation and inflammation drive phenotypic plasticity and invasion and determine therapeutic outcome (16, 50). Similarly, a pre-existing immune-active tumor microenvironment is necessary for a favorable response to ipilimumab, and potentially other ICi (51–53).

We have developed a 3D melanoma spheroid model, which recapitulates the *in vivo* tumor microenvironment and architecture (54, 55), that combined with the fluorescent ubiquitination-based cell cycle indicator (56) is a useful tool to study the microenvironment *in vitro* (57, 58). This model is being complemented constantly, e.g., by including DRAQ7 as a real-time cell death marker (59) or by applying mathematical algorithms to predict spatial and temporal patterns of cell density and cell cycle (60, 61). Due to an oxygen and nutrient gradient, melanoma spheroids segregate into a continuously proliferating subpopulation in the periphery and a G1-arrested subpopulation in the center (12). A similar phenomenon is observed in human melanoma xenografts in mice, where clusters of cycling cells are located near blood vessels and quiescent cells in central tumor zones (12). After isolating these two subpopulations from spheroids and plating them in 2D culture separately, within 24 h G1-arrested central cells recommence their cell cycle and become indistinguishable from the proliferating peripheral subpopulation (12). This supports the phenotypic plasticity model (10, 23). The cell cycle phase can also contribute to drug sensitivity (13, 62, 63) and can be targeted for cell cycle-tailored melanoma therapy (64). For example, bortezomib preferentially kills melanoma cells in the S/G2/M phase of the cell cycle (15). By contrast, cell cycle arrest can confer tolerance to drugs (14, 64, 65).

## The Role of a Slow-Cycling Subpopulation in Melanoma Therapy Resistance

Although dysregulated proliferation is a hallmark of cancer (66, 67), a quiescent or slow-cycling cell subpopulation is reported in many solid cancers, including melanoma. This slow-cycling subpopulation is a major determinant of treatment resistance to targeted therapies (68–70). Increased level of oxidative phosphorylation in slow cycling compared to normal cells (69, 71) contributes to drug resistance in many cancers including melanoma (72–74). MAPKi are predominantly effective in targeting rapidly proliferating cells, while the slow-cycling cells are not readily responsive to MAPKi (69, 75, 76). Thus, cells in the slow-cycling state or cells that switch to this state due to therapeutic stress, can evade the action of MAPKi.

**Figure 1:**
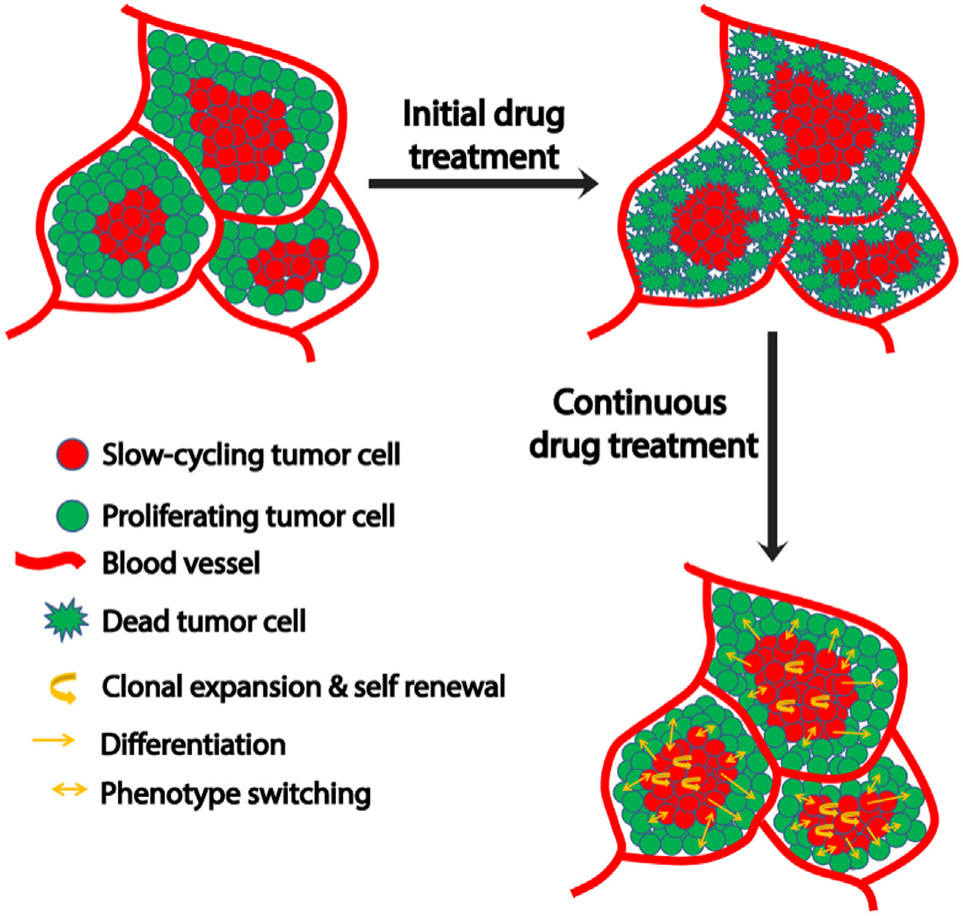
Schematic representation of microenvironment-driven dynamic heterogeneity and phenotypic plasticity as a mechanism of melanoma therapy resistance. Tumor cells close to the blood vessels proliferate, while those away from blood vessels experience hypoxia and nutrient starvation that contribute to their slow-cycling phenotype. While treatment readily targets proliferating cells, slow-cycling cells can evade drug action and survive. Upon continuous treatment, this slow-cycling subpopulation is enriched within the tumor by clonal expansion. Due to their inherent cancer stem cell-like property, they are capable of self-renewal or differentiation into a proliferative tumor cells with increased drug resistance. In addition to this, the slow-cycling cells can switch their phenotype to fast proliferating cells upon exposure to oxygen and nutrients after replacing the original peripheral fast proliferating cells that had been killed by the therapy. These phenotype-switched cells might be more drug resistant too, as they might have acquired resistance during their slow-cycling phase.

Various mechanisms are utilized by this slow-cycling subpopulation to contribute to drug resistance. First, clonal expansion of the residual slow-cycling cells, that have survived initial treatment, results in their enrichment within the tumor. A recent study suggested that these slow-cycling cells are highly aggressive with increased metastatic potential (77). Second, the slow-cycling subpopulation also displays increased cancer stem cell-like behavior (78). Consistent with the stem cell theory, in melanoma, these slow-cycling cells comprise only 0.5–5% of all tumor cells with self-renewal potential and are defined by the expression of the H3K4 demethylase JARID1B (23). In addition, JARID1B-positive cells are essential for maintaining tumor growth (23). During continuous treatment, slow-cycling cells can gain the potential to differentiate into other cell types with an increased proliferation rate and drug resistance, subsequently resulting in relapse. The cells experience a high level of “therapeutic stress,” forcing them to employ several drug resistance mechanisms. Thus, overtime highly resistant drug tolerant cells are enriched within the tumor and contribute to the highly aggressive and drug resistant nature of metastatic melanoma after relapse. JARID1B-positive cells can give rise to JARID1B-negative cells and also *vice versa* (23). This supports the phenotype switching theory which indicates the plastic nature of tumor cells that is predominantly influenced by changing microenvironmental conditions (Figure 1). In addition to JARID1B, PGC1α defines another distinct slow-cycling state in melanoma with increased treatment resistance (71, 73).

Taken together, slow-cycling cells play a pivotal role in developing therapy resistance and cancer progression. Thus, it is crucial to understand the underlying biology of the slow-cycling phenotype to improve the current therapy regimens in melanoma.

## The Role of Microphthalmia-Associated Transcription Factor (MITF) in Melanoma Plasticity and Therapy Resistance

Microphthalmia-associated transcription factor is the master regulator of both normal melanocyte and melanoma biology (79, 80). In melanoma, MITF acts as a molecular switch that determines whether the cell will differentiate, proliferate, or become quiescent with increased migratory behavior (44, 81–84). The proliferative and invasive phenotypes of melanoma cells are defined by high and low levels of MITF, respectively, and melanoma cells are capable of switching between these two states, influenced by changing microenvironmental conditions (10, 45).

Depletion of MITF can reduce proliferation through G1-arrest (42, 68, 81, 85) with increased expression of cancer stem cell markers (68, 86). In response to hypoxia, MITF expression is downregulated (87). These properties are attributes of slow-cycling JARID1B-positive melanoma cells (20), supported by a negative correlation of MITF and JARID1B/SerpinE2 (77). Thus, in response to stress, e.g., hypoxia and/or nutrient starvation, melanoma cells switch from a proliferative MITF^high^ to an invasive MITF^low^ slow-cycling phenotype. However, these subpopulations are not mutually exclusive, as within a tumor there can be MITF^high^ and MITF^low^ cells, reflecting tumor heterogeneity as discussed above. In contrast to the proliferative MITF^high^ phenotype, the invasive MITF^low^ phenotype is mainly governed by receptor tyrosine kinases (e.g., AXL, EGFR, and ERB3), WNT5A or NF-κB signaling, and the BRN2–NFIB–EZH2 axis (46, 88–90). Single cell expression analysis revealed that some MITF^high^ cells also express the gene signature of the invasive MITF^low^ phenotype (91, 92). These and other studies indicate the presence of a third subpopulation in melanoma that expresses MITF, AXL, and WNT5A simultaneously (88, 93, 94). Consistent with this, we showed by using a 3D melanoma spheroid model that indeed melanoma cells can proliferate and invade simultaneously (12). In addition, another study has shown that invasive MITF^low^ and poorly invasive MITF^high^ cells cooperate to invade into the surrounding matrix (95).

The role of MITF in drug resistance is controversial and the underlying mechanisms are yet to be understood. For instance, the presence of MITF is a marker for responsiveness to MAPKi treatment, but when MITF expression is upregulated, it can confer resistance to MAPKi (96). This might reflect the extreme end of the MITF rheostat model defined by differentiation, slow cycling (42), high PGC1α expression, and therapy resistance (20). Augmenting MITF levels in melanoma cells should switch the invasive slow-cycling phenotype to a proliferative phenotype. This would increase drug sensitivity because MAPKi predominantly act on rapidly proliferating cells. In addition, over-expression of MITF will inhibit the switching of proliferative cells to the invasive slow-cycling phenotype in response to stress by maintaining MITF levels constant. However, MITF is also reported as a driver of melanoma progression (97–99) and long-term MITF depletion induces senescence in melanoma cells and/or promotes apoptosis (81, 100, 101). Melanoma cells upregulate MITF expression to recover the loss of MAPK signaling upon exposure to MAPKi, enabling the cells to tolerate MAPKi (102). Downregulation of MITF increases the cytotoxic effects of MAPKi on melanoma cells and also reduces the acquisition of drug resistance (101, 103, 104). Upregulation of MITF has also been seen in several MAPKi acquired resistant cell lines (89). However, the same study reports that another population of resistant cell lines has lost MITF expression. MITF is downregulated in the acquired drug resistant phase and makes the cells more invasive (89). Thus, further investigation of these signaling pathways is required to determine in which combination these signaling pathways can be targeted along with the inhibition of MAPK signaling, to improve the outcomes of melanoma patients with disease relapse.

**Figure 2:**
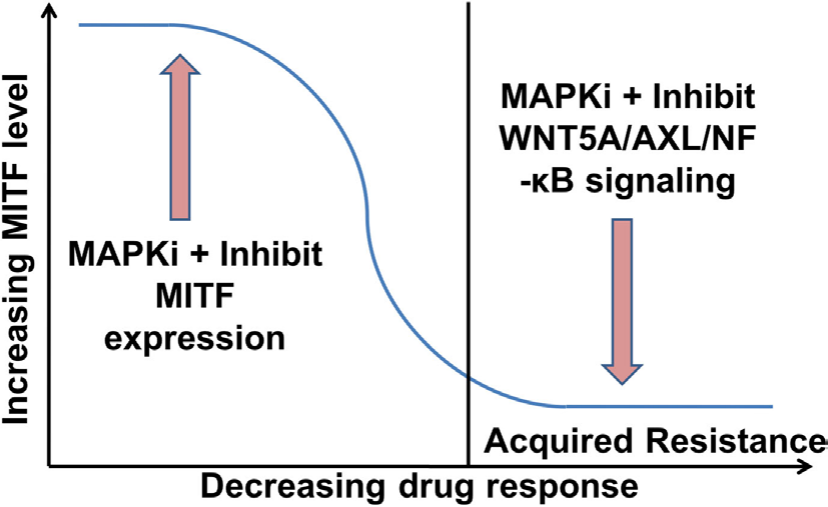
Proposed role of microphthalmia-associated transcription factor (MITF) and WNT5A/AXL/NF-κB signaling in melanoma therapy. MITF^high^ melanomas could be treated initially with a combination of an MAPK pathway inhibitors (MAPKi) and an inhibitor of MITF expression. This should increase the efficacy of the MAPKi and delay the acquisition of drug resistance (104). Once in the resistance state with low MITF levels, the therapy could comprise a combination of a MAPKi and an inhibitor of WNT5A/AXL/NF-κB signaling.

However, the situation appears to be even more complex, as in heterogeneous tumors MITF^high^ and AXL^high^ populations can co-exist (33, 102). Nevertheless, it has been shown that these subpopulations benefit from endothelin-1 in the presence of MAPKi, as inhibiting endothelin-1 signaling can effectively inhibit the growth of such heterogeneous tumors (105). More comprehensive studies are required to determine how MITF expression levels are altered in relation to the tumor’s response to MAPKi during ongoing treatment. Combination of MITF inhibitors with MAPKi should improve the efficacy of MAPKi in treating phases with high MITF expression. On the contrary, inhibitors of WNT5A/AXL/NF-κB in combination with MAPKi should improve the efficacy of MAPKi in treating phases with low MITF expression (Figure 2). Indeed, targeting AXL and BRAF/MEK simultaneously in a patient-derived xenograft model confers an increased survival advantage to the mice compared to monotherapy with either AXL or combination therapy with BRAF/MEK inhibitors (106).

## Conclusion

Tumor microenvironment-driven dynamic heterogeneity is a major determinant of drug resistance in melanoma. This is mainly exerted by regulating the level of the master regulator MITF which is the major determinant of the dynamic phenotypic states in melanoma. A moderate MITF level determines the proliferative state of melanoma which is readily targetable with MAPKi. Both low and extremely high MITF levels give rise to two distinct slow-cycling states of melanoma (e.g., MITF^low^/JARID1B-positive and MITF^high^/PGC1α-positive) with increased oxidative phosphorylation that results in treatment resistance. Thus, targeting this slow-cycling subpopulation by modulating MITF levels can be a potential strategy to overcome drug resistance in melanoma. However, MITF biology is highly complex and the downstream effects of MITF are extremely diverse (107). In addition, mechanisms that regulate MITF expression and activity are also numerous (80). Thus, modulation of MITF expression and activity can have diverse effects on melanoma cell biology. Considering the dynamic expression of MITF in response to changing microenvironmental conditions at various phases of melanomagenesis, MITF levels can be considered as a predictive marker for a suitable therapy regimen for treating a particular melanoma phase. We have developed an *in vitro* 3D melanoma spheroid model that mimics dynamic tumor heterogeneity to study the biology of microenvironment-driven tumor heterogeneity and plasticity and as these dynamic changes are difficult, time-consuming, and expensive to study *in vivo*.

## Author Contributions

NH and FA wrote the manuscript together.

## Conflict of Interest Statement

The authors declare that the research was conducted in the absence of any commercial or financial relationships that could be construed as a potential conflict of interest.
